# Examining the influences on the use of behavioural science within UK local authority public health: Qualitative thematic analysis and deductive mapping to the COM-B model and Theoretical Domains Framework

**DOI:** 10.3389/fpubh.2022.1016076

**Published:** 2022-10-20

**Authors:** Abby Moffat, Erica Jane Cook, Angel Marie Chater

**Affiliations:** ^1^Centre for Health, Wellbeing and Behaviour Change, Institute for Sport and Physical Activity Research, University of Bedfordshire, Bedford, United Kingdom; ^2^School of Psychology, University of Bedfordshire, Luton, United Kingdom; ^3^Centre for Behaviour Change, University College London, London, United Kingdom

**Keywords:** behavioural science, public health, local authority, Behaviour Change Wheel (BCW), COM-B, Theoretical Domains Framework (TDF), evidence-based practice

## Abstract

**Background:**

Behavioural science and its contribution towards improving public health is receiving increased recognition. Yet, the translation of these insights into public health practice is under-researched. This study explored the factors influencing the use of behavioural science within public health at a local authority level.

**Methods:**

Fourteen local authority staff (*n* = 13 female) in the south of England participated in semi-structured interviews, which were analysed inductively to identify key themes. These were later mapped deductively to the COM-B model and Theoretical Domains Framework.

**Findings:**

Nine themes were identified as factors that influence the use of behavioural science in local authority public health: (1) “Limited past experience,” (2) “Narrow understanding,” (3) “Perceived value of behavioural science,” (4) “Translational gap from theory-to-practice,” (5) “No protected time,” (6) “Old ways of working,” (7) “Political influence and organisational culture,” (8) “Relationships with key stakeholders,” (9) “Access to behavioural science resources”. Deductive mapping of these themes revealed that five of the COM constructs (excluding Physical Capability) and eleven of the TDF domains influenced behavioural science use, with “Social influences” and “Knowledge” being the most prominent.

**Discussion:**

Use of behavioural science within local authority public health practice is limited and inconsistent. For it to be successfully implemented, there must be an understanding of its role and value, alongside strategies to overcome a translational gap from theory to practice. Public health teams would benefit from protected time to enable application and strategies to break old habits of using a common-sense approach. System-wide buy-in, particularly related to senior leadership and system partners is needed, which would benefit from organisational and political culture change. Training opportunities, practical resources and expert in-house support should be considered a priority across public health teams.

## Introduction

Behavioural science is a multidisciplinary approach that aims to explore and understand the influence of biological, psychological, social, cultural, and environmental factors on individuals' decision-making and behaviour (1, 2). It encompasses a range of disciplines such as Psychology [study of the human mind, its functions and how these influence behaviour (3)]; Anthropology [study of the human experience concerning culture, society and linguistics (4)]; Sociology [study of the relationship between humans and their social worlds (5)]; Behavioural Economics [study of human decision-making processes (6)] and Epidemiology [study of disease frequency, distribution and patterns (7)].

Health-related behaviours have been identified as a significant determinant of public health issues currently faced both within the UK and globally (8, 9). The importance of population-level behavioural practices has become increasingly evident during the current COVID-19 pandemic where engagement in protective health behaviours and vaccine uptake has been integral in reducing infection rates and severity (10–12). Likewise, the development of non-communicable diseases that currently account for 70% of annual deaths worldwide (9) is strongly predicted by four risk behaviours: unhealthy eating, excessive alcohol consumption, non-engagement in physical activity and smoking (13, 14). In light of this, targeting health-related behaviours at a population level by drawing on behavioural science could help to tackle key public health concerns both within the UK and globally (13, 15, 16).

The contribution of behavioural science towards improving public health is now receiving growing recognition (1, 17, 18). The UK government and bodies such as Public Health England [PHE] (now the UK Health Security Agency and Office for Health Improvement and Disparities) have outlined the need to incorporate behavioural science into public health policy and practice to enable a targeted approach that minimises wasted resources (1, 8, 19, 20). A view which has been echoed globally (21, 22). In 2018, PHE and partners published a national Behavioural and Social Science strategy outlining key priorities for how this incorporation could be achieved (8). However, the extent to which behavioural science is currently used within public health practice is yet to be fully explored.

Within the UK, local authorities play an integral role in delivering the public health agenda and are expected to create, action and commission public health interventions that draw on behavioural science evidence and methodological approaches (8, 20). This need for localised integration of behavioural science has become even more important since the responsibility for public health moved from the NHS to local government in 2013 (23). In spite of this, previous research suggests that in practice this use may be limited (24–26). Local authority practitioners disclose difficulties in selecting and using behavioural science approaches due to insufficient knowledge and skills (27, 28). Inadequate reporting of disciplines, models and theories utilised in practice adds to the difficulty in ascertaining the extent to which behavioural science is used in public health practice, including commissioning, interventions, programmes and policy development (24).

An increasingly common behavioural science approach that has been used in public health research [e.g. (29–32)] is the Behaviour Change Wheel [BCW] (33, 34). The BCW is a layered framework created from a synthesis of 19 models and theories (34) that can aid the systematic development of behaviour change interventions and programmes, through an iterative process. The steps of the BCW enable the user to identify a “problem” in behavioural terms, determine the target behaviour and then identify the influences on that behaviour through a “behavioural diagnosis.” This diagnosis is performed using the COM-B model (33), which sits at the hub of the wheel. The COM-B model categorises these behavioural influences into three constructs (COM) each with two micro constructs: Capability (Psychological and Physical), Opportunity (Social and Physical) and Motivation (Reflective and Automatic) (33, 34).

To support a more in-depth interpretation of COM-B, the Theoretical Domains Framework [TDF] (35) can be used. The framework encompasses 14 domains (with skills split further into two domains) which can be directly mapped to the COM constructs: Knowledge, Cognitive and Interpersonal Skills, Memory, Attention and Decision Making, Behavioural Regulation (Psychological Capability); Physical Skills (Physical Capability); Social Influences (Social Opportunity); Environmental Context and Resources (Physical Opportunity); Beliefs about Capabilities, Beliefs about Consequences, Social/Professional Role and Identity, Intention, Goals, Optimism (Reflective Motivation); and Emotion, Reinforcement (Automatic Motivation). Insights from the COM-B model and TDF can then aid the selection of intervention strategies and behaviour change techniques (36) to facilitate behaviour change, guided by subsequent steps of the BCW (33, 34).

The BCW (33, 34) has now been recommended for use within local authority public health for the development and adaption of public health interventions and programmes. This recommendation has been supported through the provision of a step-by-step guidance document produced by PHE and the UCL Centre for Behaviour Change (37) on the use of the BCW in practice, named “Achieving behaviour change: a guide for local government and partners”.

Recent research looking at the use of behavioural science within local authority public health practice during COVID-19 (38) has found that most practitioners opted to use the BCW (34) or the COM-B model (33). However, most of these professionals were qualified health psychologists and therefore likely to have had former training and be familiar with the BCW and guidance around its use. Assessing case studies produced by public health employees further reflects this, with the majority of behavioural science informed work being conducted by those with a health psychology or behavioural science background (39–41). Outside of this, the application of behavioural science and more specifically the BCW (28, 39, 40) is limited (24). Considering local authorities' role to promote and protect population health, alongside their contribution to actioning the UK government's prevention-focused agenda (20); the need to understand and support the use of behavioural science and recommended frameworks such as the BCW within local authorities is vital.

The current study aims to understand what influences the use of behavioural science within local authority public health, using the theoretical lens of COM-B and TDF. This will provide a needs assessment for future intervention recommendations to facilitate the use of behavioural science in public health practice.

## Method

### Design

To enable an understanding of the use of behavioural science in local authority public health, a constructivist approach was adopted incorporating a qualitative study design. This was chosen to enable rich data gathering on the phenomenon of interest from the perspective of those working within this setting. Semi-structured interviews were used to explore the influences on using behavioural science in practice by local authority staff. Methodology and analysis are reported in line with the consolidated criteria for reporting qualitative research guidance [COREQ] (42).

### Recruitment and participants

#### Recruitment

Convenience sampling, led by a Public Health Principal, was used to recruit staff from a local authority within the south of England. Sixteen participants were invited to participate via email. Individuals were eligible to participate if they were adults (over 18 years) and worked within or closely alongside the public health team. Two individuals did not choose to participate. No reasons for this decision were provided.

#### Participants

The final sample (*N* = 14) included 13 females and one male, aged between 29 and 66 years (*M* = 45.57, *SD* = 10.91); 85.71% (*n* = 12) of the sample were White British, 14.29% were Black African (*n* = 2). Participants held a variety of roles across the public health team, commissioning and communications team. To the best of the authors' knowledge, none of the participants had a professional background as behavioural scientists.

### Materials

A consent form and information sheet were provided to each participant before taking part in the study. Once informed consent was sought, demographic information was collected via a survey. A semi-structured interview schedule (see Supplementary File 1) was developed via consultation of previous qualitative literature relating to the implementation of evidence-based practice in public health settings (25, 43, 44) and from the team's past experience and expertise in this area. From this, an initial list of open-ended, non-leading questions was developed by AM and AMC which was then refined through discussion with EJC. To test appropriateness and relevance, the schedule was then piloted within the population (not included in this study). No further amendments were required following this process. Interviews were used to gather data around the use of behavioural science in public health practice, and influences on its use. The researcher used prompts and follow-up questions when appropriate, to enable a participant-led interview (45).

### Procedure

Participants were provided with an information sheet via email outlining the purpose of the study and gave informed consent before taking part in an online interview using Microsoft Teams. Interviews took place from 5th March 2021 to 7th April 2021, lasting between 26 and 60 min (*M* = 41.24, *SD* = 11). All interviews were audio and video recorded; no field notes were made. They were held during office working hours and at a time when participants were instructed to work from home due to COVID-19. To the interviewer's knowledge, no one else was present whilst interviews took place.

Appropriate sample size was determined by assessing “information power”; a concept that proposes that the more relevant information held by the participants related to the phenomenon of interest, the smaller the sample size required (46). This enabled an initial approximation of sample size based on five underlying items: study aims, sample specificity, use of established theory, quality of dialogue and analysis strategy (46). This was reassessed frequently as the researcher concurrently undertook data collection and iterative analysis. Preliminary themes were regularly discussed with the research team until it was collectively agreed that the final themes were representative of the data and contribution to wider knowledge had been achieved. This approach was selected over other methods that discuss “data saturation” (47–49) due to the constructivist epistemological underpinnings of the study (50).

Participants were interviewed by the first author (AM), a Trainee Health Psychologist, who holds a BSc in Psychology, an MSc in Health Psychology and at the time of interviews was undertaking a PhD in Health Psychology with integrated competencies for Stage 2 Health Psychology training. AM has experience in qualitative interviewing and has been trained in qualitative research methods.

At the time of the interviews AM had a professional relationship with the setting, however AM did not have an established relationship with the interviewees and had only met two of the participants previously in a limited capacity. This study was completed at the start of this professional relationship to minimise bias the researcher may have had and to limit knowledge transfer to participants that may have influenced responses. AM was given the freedom to maintain limited contact with members of the organisation until all interviews had been completed. Participants were aware of AM's professional relationship with the organisation and that this was related to the provision of behavioural science expertise. Participants were made aware that the research would be used within AM's PhD thesis and Stage 2 health psychology training portfolio, however further details around AM's role and research interests were not disclosed.

After completion of the interview, participants received a verbal debrief reiterating the purpose and aims of the research and details of how participants could withdraw their data if they wished to do so. All interviews were then transcribed verbatim, of which 20% were checked for accuracy by a member of the research team (AMC).

### Ethics

Ethical approval for the current study was granted from the University of Bedfordshire's Institute for Sport and Physical Activity Research (ISPAR) ethics committee, (Reference number: - 2021ISPAR002). Throughout the study, the British Psychological Society's [BPS] Code of Human Research Ethics (51) and Code of Ethics and Conduct (52) were adhered to at all times. Informed consent was provided and a unique participant ID number was assigned to each participant to protect anonymity. Further to this, all personal information provided underwent pseudonymisation.

Data were collected and stored securely on Microsoft Teams, accessible only to the research team to uphold participant confidentiality. No risk of physical or psychological harm to participants or the research team was identified. Transcripts were shared with participants on request and redactions were made when asked for.

### Analysis

Following a constructivist approach, initial inductive analysis was undertaken via a six-step reflexive thematic analysis approach (50, 53). The interviewer (AM) coded all transcripts using NVivo 12 (54). Codes were collated and used to create preliminary themes, which were reviewed against the initial data to ensure representativeness by AM. Themes from each interview were then considered in combination and overarching themes and subthemes were identified and labelled, creating a preliminary thematic map. Data extracts, selected to support these themes and subthemes were then analysed in relation to the research question. This was then shared with the research team which lead to two iterations of themes and subthemes to ensure representativeness. Initial themes and accompanying pseudonymised data extracts were then shared with participants during a virtual meeting and feedback was sought, informing a subsequent iteration.

Based on this, a coding table (see Supplementary File 2 for final coding framework) was created and shared with the research team. Subsequent discussions lead to a further three iterations of themes and subthemes. Following this, a finalised thematic map and data extracts were determined and agreed upon by the team.

**Figure 1:**
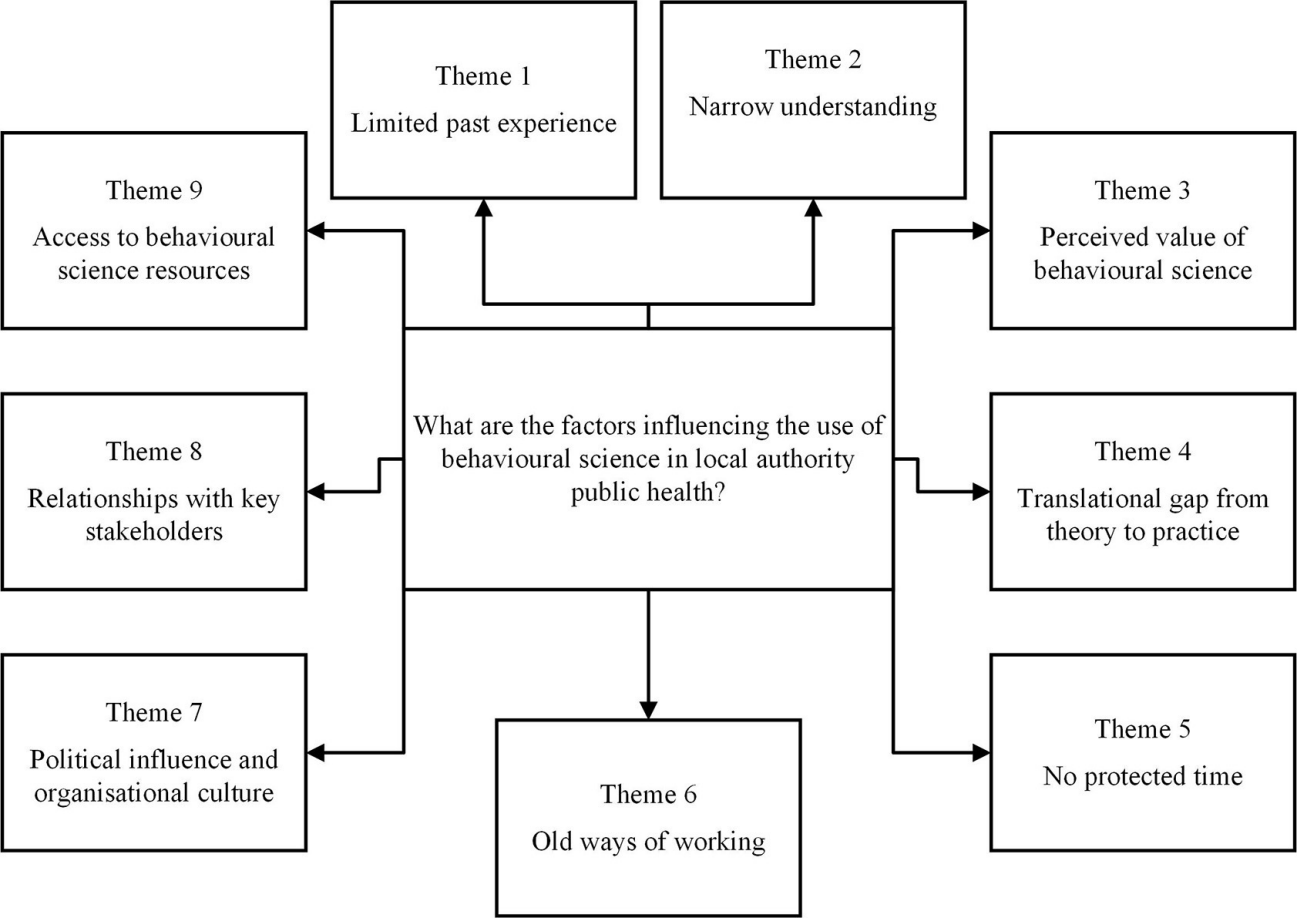
A thematic map illustrating the themes that influence the use of behavioural science in local authority public health.

Following this inductive analysis, the identified themes (see Figure 1) were deductively mapped to the TDF (35) and COM-B model (33). This was led by AM, with support from AMC and finalised in discussion with EJC. Due to the limited understanding of the phenomenon of interest, deductive mapping was carried out on the final identified themes rather than the original transcripts to ensure that any data that may have fallen outside of the TDF or COM-B domains were not overlooked.

The secondary deductive analysis also provided an opportunity to test the applicability of the TDF (35) and COM-B model (33) within this area of research and contribute towards a more comprehensive theoretical understanding of the use of behavioural science within local authority public health. This combined approach to qualitative analysis has been used previously to assess the influences on behaviour in the area of public health and implementation science (55–59).

## Results

The thematic analysis of the factors influencing the use of behavioural science within the local authority identified nine themes: (1) “Limited past experience,” (2) “Narrow understanding,” (3) “Perceived value of behavioural science,” (4) “Translational gap from theory-to-practice,” (5) “No protected time,” (6) “Old ways of working,” (7) “Political influence and organisational culture,” (8) “Relationships with key stakeholders,” (9) “Access to behavioural science resources”. See Figure 1 for thematic map.

### Theme 1: Limited past experience (COM-B = Behaviour)

#### Spectrum of use

The extent to which people reported using behavioural science previously was varied, some described using it regularly, whilst others not at all. On the whole, senior staff reported higher usage and believed this was echoed within the wider team. Many staff reported using it within past roles but that this behaviour had not translated into their current practice. Whilst others remarked that the use of behavioural science had increased as a result of the recent COVID-19 pandemic.

“*I'm trying to think of some examples. I use it lots. So, I suppose incorporating it into communications, campaigns and the way that we communicate with residents or patients as part of a project.”* (Participant 1)

“*I wouldn't say that I've used behavioural science. I suppose a lot of what I do and what I have done is based on common sense, I suppose...Yeah, 'cause I wouldn't pretend to have ever used it*.” (Participant 2)

“*I think the only time I have used it consciously in my work was a few years ago.”* (Participant 3)

“*So minimal before, this year is up with pockets of people using it.”* (Participant 4)

However, some individuals felt that their teams used elements of behavioural science in their everyday practice, but without knowing this is what it was.

“*Behaviour change is pretty fundamental to everything we do in public health, and so we are constantly thinking about bits that are behavioural science. I'm not sure that we necessarily say, OK, let's look at the COM-B model and think about this.”* (Participant 5)

“*So, umm for me behaviour science we didn't use it in a very theoretical way. But it was, we used it without knowing we were using it in our program design, in how we approached any activity.”* (Participant 6)

#### The COM-B model

For those that previously or currently use behavioural science, the COM-B model, the hub of the Behaviour Change Wheel (33, 34) was the most common approach mentioned without any priming of what would consist of behavioural science. Other models and approaches were discussed such as the Theory of Change (60), Nudge theory (61) and the FrameWorks Institute (62), yet the COM-B model was favoured across the organisation and its partners due to the structured and stepped approach it provides. However, it was felt that this use was often rudimentary.

“*Yeah, so we mainly sort of look at the COM-B model, it's probably the biggest one that we tend to use here… But again, probably we've done [it] at quite a basic level, we've not really delved deep into that.”* (Participant 7)

“*I think the COM-B model, in particular, it provided sort of a structure which was uniform across all different services, so that they were a bit more comparable…it provided that sort of stepped process that they [practitioners] could actually work through.”* (Participant 8)

### Theme 2: Narrow understanding (COM = Psychological Capability; TDF = Knowledge)

#### Behavioural science seen as synonymous with behaviour change

When asked about their understanding of behavioural science, all participants defined it as the science of understanding and changing people's behaviour.

“*My understanding is that it's the science of behaviour and what drives people to behave in certain ways, what influences behaviour, and therefore, how we might be able to influence people's behaviours by understanding the different bits that drive it.”* (Participant 5)

“*So, I suppose my understanding of behavioural science is understanding what it is that is motivating someone to do something or blocking someone from doing something and then understanding what it is you can do to make a change, whether big or small, that then means that somebody behaves differently.”* (Participant 1)

“*I think it is about understanding what makes people do what they do, and recognising that if you want to change things that you have to understand what's motivating them, and understanding things like what people say is different from what they do.”* (Participant 9)

#### Lack of clarity in knowledge

Although some individuals felt that they had an understanding of what behavioural science is, some lacked clarity in their understanding and were concerned that their knowledge lacked the appropriate depth.

“*My understanding of it is very very basic, in terms of, it's not very academic. Understanding why people do things and using that to encourage them to do what you want them to do.”* (Participant 2)

“*This won't be a very academic definition, but it's probably just about how applying science to almost how people choose to behave particularly when we're trying to influence changes in behaviour, that probably is a real dumbed down [version] of it. But it's just about where we, yeah where we're trying to change people's behaviour.”* (Participant 10)

“*I think I'm probably out of date.”* (Participant 3)

### Theme 3: Perceived value of behavioural science (COM = Reflective Motivation; TDF = Beliefs about consequences; Optimism)

#### Scepticism around behavioural science

Individuals expressed scepticism around behavioural science, specifically around its evidence base and accessibility beyond academia. These views subsequently impact the perceived value of behavioural science when used in applied practice. Alongside this, fears regarding the discipline's ethical stance and potential for unintended negative consequences were raised, resulting in a hesitancy to incorporate it into current and future work.

“*I think there is a sort of long-held scepticism amongst some people in public health, that behavioural science approaches or behaviour change approaches run the risk of entrenching health inequalities more because we focus simply on getting people to change behaviours without focusing on the structural factors.”* (Participant 5)

“*I think people possibly think behavioural science is interesting, but a bit ‘ivory tower', ‘public healthy wishy washy', you know, not very evidence-based.”* (Participant 9)

“*One of the things that slightly worries me about it [behavioural science], is it kind of gives me a feeling of manipulation and that worries me a little bit.”* (Participant 2)

“*I think some people have got a sense of behavioural science that it's just common sense.”* (Participant 11)

“*I think it is that many people don't see the value of it.”* (Participant 12)

#### Behavioural science as a valuable asset

However, many participants discussed how behavioural science could be a valuable addition to their work, enabling a more informed approach to intervention development, a clearer overview of projects and space to reflect.

“*We wouldn't just be doing it because XYZ has done it and it works. You know, we'd be thinking a lot more about impact, and the outcomes that we're looking for.”* (Participant 13)

“*Well, I think by using them [behavioural science approaches] you have more of a holistic approach to interventions. Rather than focusing on let's say a problem, you can see how everything is integrated and also it helps with setting realistic expectations and also in project design.”* (Participant 6)

“*…something that's really useful about a behavioural science approach where you're looking at a framework, or more than one framework sometimes, is it gives you a chance to sit down and reflect on what you're doing and understand where something is missing.”* (Participant 5)

#### Demonstrating the value

Participants expressed the importance of demonstrating the value of using a behavioural science approach to colleagues and decision-makers to address scepticism and associated concerns. This needs to be evidenced clearly and simply through hard figures and relevant examples if buy-in to this approach is to be generated throughout the organisation.

“*I'd probably say from a commissioning point of view, people generally like quite hard figures about it [what impact using behavioural science makes], so it's like look at this and this is what happened to performance in terms of engagement with a particular group, directly attributed to that.”* (Participant 11)

“*It's the kind of ‘so what' factor, and having more examples of that because I think in a local authority it's about bringing it back to the fundamental of what difference it's made for those residents or that population….and it's about the local authority being able to see that.”* (Participant 10)

“*We've also got to show something for why we're advocating for this… it's gotta be well coordinated and well done. Done properly so that it's very evident that- look this is the evidence, this would work.”* (Participant 13)

### Theme 4: Translational gap from theory-to-practice (COM = Psychological Capability; TDF = Knowledge; Skills (cognitive and interpersonal))

#### Translating learnings into practice

One factor that participants felt inhibits the use of behavioural science is the presence of a theory-practice gap. Even when training has been previously received this has not translated into meaningful changes in practice and was not seen as a long-term solution to embedding behavioural science.

“*Again, I think it's probably that going from the theory to the practical, is probably the biggest challenge that I've found.”* (Participant 7)

“*There was loads of interesting stuff from it [previous training]. It's just I think that putting it into practice is the hard part.”* (Participant 14)

“*I think especially if you're implementing something like the COM-B model and the Behaviour Change Wheel, a day's training is not, it's not enough, you need to be constantly looking and exploring how it can actually be used in practice.”* (Participant 8)

#### Being too academic limits understanding and application

An explanation for this theory-to-practice gap was that how material has been received was overly academic. This did not enable the easy translation of the taught content to practical application. The way previous training had been delivered made it difficult to comprehend or see how it would be implemented. Furthermore, individuals felt uncomfortable admitting that they did not fully understand the training being received, or needing more concrete skill development to be able to apply the knowledge in practice.

“*I've been to a number of presentations…And they were frightfully academic and lots and lots of theory and the irony was that I didn't feel that what they were saying helped me change my behaviour because it was too abstract. Rather than lots of real examples that I could think I can do that.”* (Participant 12)

“*Where obviously they're [trainer] the expert on it, they work on it pretty much every day, so to them they're like oh this bits really easy, but you're looking at the model thinking that's just gone completely over my head. Um but you might not want to say anything ‘cause you don't want to be the only one that doesn't understand.”* (Participant 14)

Participants discussed how future learning material needs to be made applicable and relevant to their roles, through case studies and real-life examples. This would facilitate understanding and translation into practice.

“*Yeah, it needs to be made so people can see the relevance of it for their roles. Cause otherwise, they might think oh well that's really interesting, but then not be able to apply it to their own roles. So, what we need to be able to do is make them see it's relevant to what they do.”* (Participant 4)

“*…so simplifying and really emphasise on how it works. That's why I like case studies, I think that for us, for all of us to understand, through case studies and proper examples we can easily embed it, but there is a huge knowledge gap.”* (Participant 6)

### Theme 5: No protected time (COM = Physical Opportunity; Social Opportunity; TDF = Environmental context and resources; Social influences)

#### Too busy

An overarching factor that may be contributing to many of the other identified barriers, is a lack of time to learn, digest and implement behavioural science.

“*…at the moment the challenge is time. Fundamentally, absolutely, there just isn't time to sit down and think how can I do this in X way?”* (Participant 5)

“*I think that at the moment the biggest challenge is that everyone's so busy it's just trying to fit it in [and] get people to see that actually no, probably in the long run it'll probably save time, but it's just that initial investment of time.”* (Participant 7)

#### Unrealistic expectations

The lack of time to incorporate behavioural science was further impacted by a perceived pressure to complete work quickly, leading to a potential disconnect between how long projects should take and how long individuals are expected to complete them within.

“*…if you're going to properly think about doing it, you know, in a staged way, it takes more time. Which from other people's perspective doesn't necessarily always work.”* (Participant 3)

“*So sometimes it's the speed with which things need to be done, and it's difficult to take the time to think it through and think how you might do it differently.”* (Participant 2)

#### Impact of time pressures

These time pressures have further knock-on effects such as a reduction in motivation and headspace to consider the use of behavioural science and less emphasis on thorough evaluation to measure impact and identify areas for improvement.

“*When we're all so busy and you've got loads of different plates spinning and you're trying to remember everything, you just want to get the campaign done. You haven't necessarily got the time to sit down and think, oh, this is a good campaign, but how can I make it better by adding in behavioural science.”* (Participant 14)

“*But for me, behavioural science is also that bit about measuring what impact you're having and identifying where things have worked, where things haven't worked, and where things can be improved. That's something I would like to do more of if we only had the time.”* (Participant 5)

“*Um ‘cause when you haven't got the headspace or when you're trying to fit it in at like 9:00 o'clock at night, um, your brain just can't take it in can it?”* (Participant 1)

#### Allocated time needed

Many participants expressed a need for some protected time to be able to learn more about behavioural science and hone their skills and understanding. This would ensure they were up to speed on relevant materials such as reading published behavioural science guidance and resources. It was suggested that protected time may not be deemed important for those working in public health due to the slow pace at which the field usually changes. However, participants felt it was essential when trying to adopt a new way of working.

“*…the need for kind of protected time to learn may not seem as much of a priority for our particular field, perhaps. So, it's important when things like new theories around behavioural science emerge that we've got the time to sort of absorb them and understand them and read them and digest them and think about how we can use them.”* (Participant 5)

“*…with every will in the world I really want to read them [BPS behavioural science resources], but I can't…so having some protected time in a CPD session…would be really helpful.”* (Participant 1)

*I don't think you can rush this stuff because it's a new way of thinking and you've gotta take your time and get it right.”* (Participant 4)

### Theme 6: Old ways of working (COM = Automatic Motivation; Psychological Capability; Reflective Motivation; TDF = Reinforcement; Emotion; Behavioural regulation; Goals; Beliefs about consequences; Social/ Professional role and identity)

#### Slipping back into old ways

One factor that appears to heavily influence the use of behaviour science is habit. Participants felt that many people express the intention to use behavioural science but this often does not come to fruition, with individuals slowly falling back into existing routines.

“*When you get people in a room and you help facilitate their thinking, they're like oh and what about this? And what about that? But then when they get back to the office, again the normal, however it has to be done comes in and all of that goes out the window.”* (Participant 1)

“*…what we need to do is make sure that keeps being reinforced, and I suppose you know, we can slip back into old ways quite quickly.”* (Participant 4)

#### Becoming second nature

Participants stated that the use of behavioural science needs to be continually reinforced if it is to become part of everyday practice. The ultimate desire is for its use to become an automatic process within the team and the standard way of working.

“*…so to try and get it really embedded in people's thinking, so they don't even think of doing it a different way”* (Participant 1)

“*…we need people to get into the habit of it just being second nature.”* (Participant 9)

“*Um so, make it more second nature I suppose, and that only comes with keeping going over it and learning more about it and just having it there on the agenda.”* (Participant 14)

#### Changing practice can be emotive

Individuals' perception of behavioural science and how this links with their practice can lead to emotionally driven responses that can act to inhibit or promote the desire to adopt this new way of working. Participants discussed how the introduction of behavioural science into everyday practice could lead to feelings of being patronised or criticised, and that for some having to reflect on their current practice and identify a need for change could be difficult.

“*I think that some people, just, it gets their backs up when they feel as though they're being told something that they feel is common sense”*. (Participant 11)

“*If the message fundamentally, is you need to do something differently, you are criticising what they were doing before and saying what you were doing before wasn't effective, it wasn't good. Therefore, you know, you're not doing your job properly. That's the implicit message.”* (Participant 12)

“*I think the bit about the personal development though as well, like the sitting down looking at yourself can be quite difficult to do.”* (Participant 5)

Yet, if the proposed changes are perceived positively this can result in individuals embracing it as an opportunity to improve practice linked to positive emotion, rather than seeing it as a threat to the current status quo.

“*I think it's really refreshing. I think it can get you excited about what you're trying to do again rather than it just feeling like ohh we've been here before. We're gonna come up with the same ideas and go round in the same circles.”* (Participant 1)

### Theme 7: Political influence and organisational culture (COM = Social Opportunity; TDF = Social influences)

#### Influence of political lens on council culture

The culture of the council was described by participants as being strongly influenced by politics. Subsequently, policies and methods of working were said to be constructed through a political lens which heavily influenced staff behaviour and outcomes.

“*Local authorities' policy is not based on evidence; it's based on political ideology.”* (Participant 12)

“*Conservative government and their general approach errs more toward people should take responsibility for their own actions and so what they have drawn from behavioural science and nudge has been more about that aspect of behavioural science approaches. I suspect if we had a different political leadership, we would see a different reaction and different responses, so it's all kind of intertwined…I mean public health is politics, isn't it?”* (Participant 5)

“*So, I see the value in us being in the local authority, but I don't know. I just see that there's a lot of politics and that in itself prevents some of these things from happening.”* (Participant 13)

#### Politically driven impacts

Political needs were seen to dictate the ability to implement behavioural science approaches due to concerns regarding the council's reputation. Consequently, this leads to risk aversion, particularly when individuals want to implement a new approach such as behavioural science where uncertainty may exist around outcomes.

“*And they tell you, its government money, it's public money…we've got to show why we're doing this and maybe sometimes the focus is too much on that and we actually lose sight of what we're actually trying to do.”* (Participant 13)

“*Getting sort of sign-off in a council can either be impossible or just takes a really long time. So, it's that kind of political environment I think makes it quite difficult. And also, if you're a council that is risk-averse. Then you don't get to do those projects in the same way as a different council.”* (Participant 14)

“*…sometimes it's quite hard to show things that work and people really want them to work, so politically it's not great if you do evaluate something and then it doesn't work, that can be a real problem for people, which is a shame.”* (Participant 5)

#### Cultural change needs to be driven from the top

In light of this, many of the participants suggested that there is a need for a culture shift, not just at a local authority level but nationally, and that part of this culture shift should be to incorporate behavioural science into everyday practice.

“*It kind of needs a massive culture shift at a national level as well as at a local level, where politicians are really bought into.”* (Participant 5)

“*But the concept is the organisational culture being focused on whatever it is, and it's got to be something which is tangible…What's the culture that we want to engender? And so, the chief executive, executive directors, then the other directors, assistant directors have got to be totally bought into that, and believe it and behave that way themselves.”* (Participant 12)

There was a common consensus across participants that this culture shift needs to be driven by senior management and those in positions of authority both across the local authority and the wider public health sector.

“*So, getting people higher up the organisation to be promoting it and talking about the value of it…there's nothing like a leader or a politician talking about something to make other people want to take part.”* (Participant 2)

“*So, I think that has to start at the top. They need to understand the concepts and how it works and then it will have a trickle down effect on my work.”* (Participant 6)

“*I think it has to come from the top. Very much from the top. Because those of us who beli- it's like preaching to the converted. We understand that, we know what it is, we understand, but at the end of the day, we will not be the ones making the ultimate decisions.”* (Participant 13)

### Theme 8: Relationships with key stakeholders (COM = Social Opportunity; TDF = Social influences)

#### Interdependence

It was emphasised by participants that public health teams do not work in isolation and are very dependent on other teams and organisations to create and implement projects.

“*I mean everything we do in public health is, well, not everything, but so much is so reliant on partnerships and influencing people.”* (Participant 1)

“*…because in public health we deliver some of the intervention, but usually we need to work with a lot of other people to get things done successfully, you know, it's that whole thing of public health needs to be everybody's business for it to have proper change.”* (Participant 5)

And although individuals believe that collaborative working is not only expected within public health but is essential to successfully integrate behavioural science into everyday practice, it is acknowledged that currently teams and systems don't always work in this way.

“*Yeah, we don't always work quite so joined up. We do have some quite siloed systems. So, I think that is a challenge.”* (Participant 10*)*

#### Working relationships

Participants discussed how difficulties in collaborative working stem from challenging working relationships. This often involves conflicting priorities, a lack of understanding of each party's role and disputes over decision-making.

“*Everyone's always got different opinions and priorities, so you know, me and [a colleague] might have pushed the provider to do comms whereas the commissioner might have said, well, we need to get them focused on this other bit of the contract.”* (Participant 14)

“*There is not a good relationship between the NHS and local authorities. There are a number of reasons behind that, partly the NHS just does not understand local authorities, and I'm not sure local authorities really understand the NHS. There are times when they can work together on some things, but scope for improvement is phenomenal.”* (Participant 12)

“*…cause the community's really great and we want to work with them much more closely. Some of the problems are these tensions between who's allowed to work with them.”* (Participant 9)

#### Getting others to engage

Participants talked about the difficulties in getting partners to engage in projects and the subsequent frustration this causes. It was felt that even if participants proposed the use of behavioural science, they could not enforce this, but only suggest. Therefore, to enable a social/cultural environment in which collaborative working can be achieved, these underlying factors would need to be addressed and overcome.

“*Or you're asking partners to come up with some ideas for actions. Actual actions that they're actually going to do. Umm and that's the tricky bit because nobody wants to commit to doing anything when everyone is so time pressured and they think it's going to be extra work.”* (Participant 1)

“*So, for my work, I again would just like the provider to utilise it a bit more 'cause we don't have control over what they do, we can just suggest things. So, we can keep suggesting have you thought of this? Have you thought of that? But it doesn't mean that they'll actually do it, so that's quite frustrating.”* (Participant 14)

“*So, we really want to be engaging a lot of our NHS colleagues in primary and secondary prevention… And, it was hard, it has been challenging to engage our NHS colleagues in doing some of that anyway. It's often very difficult, I mean, this isn't new. This is a long-standing problem.”* (Participant 5)

### Theme 9: Access to behavioural science resources (COM = Psychological Capability; Physical Opportunity; Social Opportunity; TDF = Knowledge; Skills (cognitive and interpersonal); Environmental context and resources; Social influences)

#### Training

To enable the use of behavioural science in practice participants suggested further training to help upskill the workforce. However, it was highlighted that due to high staff turnover this needed to be a consistent approach with training being offered at regular intervals. It was suggested that E-learning modules could help make this training more accessible.

“*…it's about how we support people to understand that information in the fullest way possible. I think training, as much as face-to-face training is always the answer to everything, but I really do think that some form of session, to inform people about it, but equally to help them realise actually in their own context what that might look like, and I think that only comes from discussion in that type of environment.”* (Participant 11)

“*If you were looking to train those teams up, the problem you have is that the staff turnover can be quite high in those services, so it needs to be a constant sort of training, it needs to be part of induction processes…and then making sure they're doing regular top-ups.”* (Participant 8)

“*I think it would be useful if there was like some kind of E-learning module.”* (Participant 13)

#### Checklists to guide practice

Alongside training, participants felt that having a step-by-step checklist resource that could guide the use of behavioural science in practice would be helpful. It was highlighted that this needs to be straightforward and be aimed at making the translation of theory and the evidence-base as easy as possible for those using it in everyday public health practice.

“*I suppose you could have a checklist that was like: you're thinking of a new project, have you thought about this, this and this?... It doesn't have to be all of it, you could just pick out maybe like one of these principles or something from the COM-B model.”* (Participant 14)

“*…having more like a checklist to help you think what you should be thinking about. Like very simple, did you think about A, B, C.”* (Participant 6)

#### Requiring support from behavioural science experts

The need for additional ongoing support to integrate behavioural science was raised by many participants as individuals were aware that their expertise was not in this area. This was discussed in relation to the need for professional support to help guide practice.

“*What would be really nice is kind of ongoing support, so whether that's through action learning sets, where people have got access to someone who can just kind of go, yeah, I think you're on the right track.”* (Participant 11)

“*We're not experts so we don't know what messages will work and resonate. It's a bit of a guessing game at the moment, so I think it would be useful to have some input into that.”* (Participant 7)

“*It's just someone talking you through in a practical approach I think is what is actually needed. So how you would actually in practice put it into place.”* (Participant 8)

There is a preference for this support to be in-house, as this person would understand the organisation and the parameters in which people are working. This would be preferable to external consultants. It was felt that this support could facilitate changes in practice within the team.

“*…to have someone in-house that understands your organisation as well is really helpful. Because sometimes you can have someone that's an expert outside, but if they don't understand the boundaries you work within and the costs that we work within, then it's quite tricky.”* (Participant 4)

“*I think that having a person in our team who specialises in behavioural science that would provide support… So, whenever we're creating a new project or we are implementing a project, somebody, more like an external consultant, but they're not external, they're part of our team that we can go and ask questions, and I think by having a dedicated person in our team we will learn by osmosis.”* (Participant 6)

## Discussion

The current study explored what influences the use of behavioural science within local authority public health practice. Using inductive thematic analysis nine themes were identified: (1) “Limited past experience,” (2) “Narrow understanding,” (3) “Perceived value of behavioural science,” (4) “Translational gap from theory-to-practice,” (5) “No protected time,” (6) “Old ways of working,” (7) “Political influence and organisational culture,” (8) “Relationships with key stakeholders,” (9) “Access to behavioural science resources.”

Deductive coding of these themes to the COM-B (33, 34) and TDF (35) revealed that five of the COM constructs (excluding Physical Capability) and 11 of the TDF domains influence the use of behavioural science in local authority public health: Psychological Capability (Knowledge, Skills (cognitive and interpersonal), Behavioural regulation); Physical Opportunity (Environmental context and resources); Social Opportunity (Social influences); Reflective Motivation (Beliefs about consequences, Optimism, Goals, Social/ Professional role and identity) and Automatic Motivation (Reinforcement, Emotion). “Social Opportunity” and “Psychological Capability” were the most prominent COM constructs. The most common TDF domains were “Social influences” and “Knowledge” (see Supplementary File 2 for full coding framework). This indicates that while there are many influences on the use of behavioural science, the social environment in which the public health team operates and a lack of understanding regarding behavioural science most strongly impact the use of these approaches within everyday practice.

Overall, the use of behavioural science within local authority public health was found to be limited and inconsistent, in line with previous findings (24–26). However, attempts to use behavioural science were reported to have increased since the COVID-19 pandemic. This supports previous qualitative work (38, 63) which reported that public health teams' desire to use behavioural science and wider research evidence within their practice had increased significantly since the beginning of the pandemic. This increased interest is thought to be due to the greater value placed on scientific evidence during this time (63).

The COM-B model (33) was the most commonly used approach mentioned when describing behavioural science. Although the COM-B model sits within the BCW (33, 34) this suggests that this recommended framework is not being utilised in its entirety. Given that the BCW provides a step-by-step approach to intervention development, its partial use may lead to inappropriate choices around intervention components, which could subsequently impact outcomes (64, 65). Therefore, gathering a further understanding of what may inhibit the use of the BCW is important, particularly as the interest in behavioural science approaches continues to grow across public health (38).

Overall participants demonstrated limitations around their understanding of behavioural science approaches leading to a theory-practice gap, with practitioners unable to action prior learning as the resources available were “too academic” and lacked relevance to their day-to-day practice. Similar findings were reported by Byrne-Davis et al. (38) where this gap was attributed to the pressure of the pandemic. However, the current findings suggest this is a more global issue that extends beyond periods of additional pressure and is more reflective of a need to improve knowledge and skills around the application of behavioural science. Alongside this, it indicates a need to reconsider dissemination techniques of behavioural science evidence to make theory and resources accessible to those who play a key role in actioning behavioural science insights and facilitating real-world behaviour change (20, 66).

Participants felt that this theory-practice gap could be bridged through the use of contextualised examples, such as case studies, and via the provision of training, guidance tools, checklists and in-house expert support. The need for tools and support to guide the implementation of new knowledge into practice and build capacity has been identified as a facilitator across health systems and local authorities (44, 63, 67–71), and was outlined as a key priority within the national strategy produced by PHE and partners (8). In-house expert support and collaborative partnerships with universities have also been recommended by the Local Government Association (72). Such tools and expert guidance that have been used previously to support the uptake of evidence-based approaches were found to increase self-efficacy, understanding and perceived relevance of information (73, 74).

As well as increasing understanding, the use of such guidance tools and resources has also been reported to increase perceived efficiency (74), which could help address the barrier of time constraints raised by participants. Insufficient time to implement evidence-based practice has been reported across public health settings (43, 69, 75, 76) and is considered to significantly inhibit the uptake of new approaches (25, 77, 78). Participants suggested that providing protected time could help to overcome this and would enable teams to digest and implement new knowledge and skill development. However, buy-in from colleagues and senior management is integral to being afforded the time and space to use these approaches.

Participants felt this buy-in could be achieved by demonstrating the value of using behavioural science through clear evidence of its impact. A difficulty here is synthesising evidence from a number of disciplines that may fall within behavioural science. Given the evidence in this paper that the COM-B model (33) is the most acknowledged approach, alongside the promotion of the wider Behaviour Change Wheel (33, 34) in the PHE guide to achieving behaviour change (37), it may be most beneficial to focus on the efficacy of this particular approach. There are now an array of academic papers and case studies exhibiting the use of the BCW and its components (40, 41, 58, 79–81). However, there is currently no evidence synthesis around its use within public health. Providing such a synthesis could help showcase the value of this approach to public health practice and increase evidence of the value of its use to achieve positive outcomes.

However, the limitations around academic evidence alone was raised in a subsequent theme, where participants discussed the impact of politics on behavioural science use in everyday practice. This reflects previous research within public health commissioning, where return on investment and political needs were proposed to overshadow scientific evidence (25, 43). Tensions between public health and political agendas have been highlighted within the wider literature (23, 75, 82). Elected members who are responsible for key public health decision-making (27, 28, 43), are said to also be driven by political ideology and economic considerations which can impact public health practice (23, 28, 43). It was suggested that to fully enable the use of behavioural science a culture change would need to occur, lead by both politicians and senior leaders.

Evidence supports that leaders have the ability to shape and adapt the culture of their organisation and its practices (67, 70, 83–85). Specifically, Curtis et al. (25) found that when elected members were supportive of using behavioural science, that enabled its use in practice. Therefore, having elected members and senior employees of the organisation who are fully bought into using behavioural science approaches are fundamental to successfully embedding it in everyday practice. However, culture change within public health is often difficult due to wider bureaucracy (86, 87), which also highlights a need to advocate for its inclusion at a national level.

Outside of leaders, participants also emphasised how the interconnectedness of their work with colleagues and external partners influenced their ability to utilise behavioural science. This suggests that the social norms around its use need to be extended beyond the public health team itself and that resources such as training, guidance and support need to be offered more widely. Challenges around engaging colleagues in the use of behavioural science and having its incorporation widely accepted were also highlighted as a barrier by behavioural scientists working within public health during the pandemic (38). This indicates that a collaborative whole systems approach needs to be taken if behavioural science is to be used effectively within public health practice.

### Implications

The current study extends existing knowledge regarding the individual, group and organisational level factors that are influencing the use of behavioural science within public health. It provides further support for the impact of political and organisational climates on public health practice and highlights the importance of senior level support alongside the allocation of protected time, further training and resources. The findings also indicate a need for closer collaboration between public health practitioners and academic colleagues, in the form of partnerships or through in-house employment opportunities. Developing targeted intervention strategies that encompass these factors could contribute towards increased incorporation of behavioural science within local authority public health practice.

However, the findings demonstrate that public health teams do not work in isolation and the use of behavioural science in practice is dependent on many other actors within the system. In light of this, future qualitative research is needed to explore the perspectives of all stakeholders involved in the implementation process. Drawing on the current findings, this could include local authority senior management, elected members, service providers, fellow local authority departments and external partnering organisations. Establishing this comprehensive understanding would ensure that any interventions that target the use of behavioural science are considering the context and environment in which public health practitioners are operating.

### Limitations

Although previously used to explore implementation of evidence-based practice (25, 88–91), the current findings indicate that the COM-B model (33) and TDF (35) may not provide sufficient depth of understanding regarding the impact of organisational and contextual factors upon behavioural science use in local authority public health settings. Retrospectively, using the COM-B and TDF in combination with an organisational behaviour theory or model could have provided a more nuanced understanding of these factors and their relationship to behavioural science use. In light of this, future work in this area should consult research in organisational behaviour, culture and climate to contribute towards understanding and intervention development, alongside behaviour change models and frameworks such as the Behaviour Change Wheel (33, 34).

Due to the nature of qualitative research, the current findings cannot be generalised to the wider public health population. This is further limited by the inclusion of a single local authority, however, as the participants had worked across various organisations and drew on this experience during interviews, the findings could indicate some transferability across the local authority public health system (92). To gather further insight into this, subsequent research should be undertaken within other local authorities to establish themes that are present more broadly.

## Conclusion

The use of behavioural science within local authority public health practice is limited and inconsistent. For it to be successfully implemented, there must be a provision of accessible training, guidance and expert support to improve knowledge and skills, reduce the translational gap from theory to practice and change habitual ways of working. However, the use of behavioural science requires further system-wide buy-in, support and collaborative working to ensure that behavioural science is actioned in practice. Senior leadership have a key role in enabling this through organisational and political culture change. This evidence provides a template for key considerations for future interventions to widen the use of behavioural science, and specifically the BCW, in public health practice.

## Data availability statement

The raw data that supports the conclusions of this article will be made available upon request after the publication of AM's PhD thesis.

## Ethics statement

This study was reviewed and approved by the Institute for Sport and Physical Activity Research (ISPAR) Ethics Committee, University of Bedfordshire. The participants provided their written informed consent to participate in this study.

## Author contributions

AM, EJC, and AMC conceived and designed the study. AM collected the data and wrote the first draft of the manuscript. AM and AMC analysed the data, with contributions from EJC. All authors contributed to revisions of the manuscript and approved the final manuscript.

## Funding

This research was part of AM's PhD studentship secured by AMC from local authority funding and registered with the University of Bedfordshire (RES20108).

## Conflict of interest

AM had a professional relationship with the setting of interest. However, for this study AM was acting as an independent researcher. The remaining authors declare that the research was conducted in the absence of any commercial or financial relationships that could be construed as a potential conflict of interest.

## Publisher's note

All claims expressed in this article are solely those of the authors and do not necessarily represent those of their affiliated organizations, or those of the publisher, the editors and the reviewers. Any product that may be evaluated in this article, or claim that may be made by its manufacturer, is not guaranteed or endorsed by the publisher.
