# Supplementary material for: Examining the influences on the use of behavioural science within UK local authority public health: Qualitative thematic analysis and deductive mapping to the COM-B model and Theoretical Domains Framework
Source: Front Public Health. 2022 Oct 20;10:1016076. doi: 10.3389/fpubh.2022.1016076 (PMC9632167; doi:10.3389/fpubh.2022.1016076)
Supplement: Supplementary file 1 [file Data_Sheet_1.PDF]

Supplementary file 1- Interview schedule for understanding the influences on the use of behavioural science in local authority public health.

Interview schedule

1.What is your understanding of behavioural science?

1a) If aware of behavioural science: Where has your understanding of behavioural science come from?

Prompts: Has this come from workshops/training/ own research? During your time at your current organisation (e.g., council) or via previous role / employer?

1b) If heard of but not aware of behavioural science: Where have you heard this term used before?

1c) If totally not heard of it: Give an overview

'Behavioural science is an umbrella term that includes a number of disciplines such as Psychology, Sociology and Economics that deal with human actions. In essence what behavioural science aims to do is look at the biological, social, psychological and environmental factors that influence an individual's thoughts, preferences, decision making and behaviour. This information can then be used to create behaviour change by considering how these factors influence an individual's behaviour and then subsequently developing strategies and interventions to help reshape this behaviour in the desired way. For example, helping people to quit smoking or increasing their engagement in physical activity.'

2. What is your experience of using behavioural science?

Prompts: Where did you use this? How did you use it? What was the outcome?

3. What were the positives of using behavioural science in this work?

4. What made it challenging?

5.What do you feel could help overcome these challenges?

6. How do [would] you currently access information related to behavioural science?

7. How would you like to be able to access information and support related to behavioural science?

Prompts: What format would you like to access this in?

8. How do you feel behavioural science could be best used within your work/ organisation?

Prompts: Who would be involved in this? How would you see this working in practice?

9. What would help to facilitate this use of behavioural science within your work?

10. Do you think behavioural science should be embedded into everyday practice?

10a) How can this happen?

11. Where would behavioural science be most effective within your organisation?

Prompts: Why do you believe it would be most effective in this area? What other areas might benefit from behavioural science?

12. Are you aware of the behavioural science guidance documents and ‘roadmaps’ that have been produced by the British Psychological Society?

12 a) If yes: How have these documents been used?

12 b) If no: How could we best disseminate these sorts of documents to you?

13. How could the use of behavioural science improve your work and organisation?
