# Supplementary material for: Examining the influences on the use of behavioural science within UK local authority public health: Qualitative thematic analysis and deductive mapping to the COM-B model and Theoretical Domains Framework
Source: Front Public Health. 2022 Oct 20;10:1016076. doi: 10.3389/fpubh.2022.1016076 (PMC9632167; doi:10.3389/fpubh.2022.1016076)
Supplement: Supplementary file 2 [file Data_Sheet_2.PDF]

Supplementary file 2- Full coding framework

**Theme**	**COM-B Construct(s)**	**COM-B Micro-construct(s)**	**TDF Domain**
Limited past experience	Behaviour		
Narrow understanding	Capability	Psychological Capability	Knowledge
Perceived value of behavioural science	Motivation	Reflective Motivation	Beliefs about Consequences; Optimism
Translational gap from theory-to-practice	Capability	Psychological Capability	Knowledge; Skills (cognitive and interpersonal)
No protected time	Opportunity	Physical Opportunity; Social Opportunity	Environmental Context and Resources; Social Influences
Old ways of working	Motivation; Capability	Automatic Motivation; Psychological Capability; Reflective Motivation	Reinforcement; Emotion; Behavioural regulation; Goals; Beliefs about consequences; Social/ professional role and identity

Political influence and organisational culture	Opportunity	Social Opportunity	Social Influences
Relationships with key stakeholders	Opportunity	Social Opportunity	Social Influences
Access to behavioural science resources	Capability; Opportunity	Psychological Capability; Physical Opportunity; Social Opportunity	Knowledge; Skills (cognitive and interpersonal); Environmental Context and Resources; Social influences
